# Decreased Autophagy Contributes to Myocardial Dysfunction in Rats Subjected to Nonlethal Mechanical Trauma

**DOI:** 10.1371/journal.pone.0071400

**Published:** 2013-08-19

**Authors:** Jie Wang, Keyi Lu, Feng Liang, Xiaoyu Li, Li Wang, Caihong Yang, Zi Yan, Suli Zhang, Huirong Liu

**Affiliations:** 1 Department of Physiology, Shanxi Medical University, Taiyuan, Shanxi, P. R. China; 2 Department of Neurology, First Hospital of Shanxi Medical University, Taiyuan, Shanxi, P. R. China; 3 Department of Nuclear Medicine, First Hospital of Shanxi Medical University, Taiyuan, Shanxi, P. R. China; 4 Cardiothoracic Surgery, Steel General Hospital of Taiyuan, Taiyuan, Shanxi, P. R. China; 5 Molecular Biology Laboratory, Shanxi Tumor Hospital, Taiyuan, Shanxi, P. R. China; 6 Department of Pharmacology, Shanxi Medical University, Taiyuan, Shanxi, P. R. China; 7 Department of Pathophysiology, Capital Medical University, School of Basic Medical Sciences, Beijing, P. R. China; 8 The Key Laboratory of Remodeling-related Cardiovascular Diseases, Capital Medical University, Ministry of Education, Beijing, P. R. China; Thomas Jefferson University, United States of America

## Abstract

Autophagy is important in cells for removing damaged organelles, such as mitochondria. Insufficient autophagy plays a critical role in tissue injury and organ dysfunction under a variety of pathological conditions. However, the role of autophagy in nonlethal traumatic cardiac damage remains unclear. The aims of the present study were to investigate whether nonlethal mechanical trauma may result in the change of cardiomyocyte autophagy, and if so, to determine whether the changed myocardial autophagy may contribute to delayed cardiac dysfunction. Male adult rats were subjected to nonlethal traumatic injury, and cardiomyocyte autophagy, cardiac mitochondrial function, and cardiac function in isolated perfused hearts were detected. Direct mechanical traumatic injury was not observed in the heart within 24 h after trauma. However, cardiomyocyte autophagy gradually decreased and reached a minimal level 6 h after trauma. Cardiac mitochondrial dysfunction was observed by cardiac radionuclide imaging 6 h after trauma, and cardiac dysfunction was observed 24 h after trauma in the isolated perfused heart. These were reversed when autophagy was induced by administration of the autophagy inducer rapamycin 30 min before trauma. Our present study demonstrated for the first time that nonlethal traumatic injury caused decreased autophagy, and decreased autophagy may contribute to post-traumatic organ dysfunction. Though our study has some limitations, it strongly suggests that cardiac damage induced by nonlethal mechanical trauma can be detected by noninvasive radionuclide imaging, and induction of autophagy may be a novel strategy for reducing posttrauma multiple organ failure.

## Introduction

Mechanical trauma, such as that induced by natural disaster, athletic sports, war, and motor vehicle crashes, represents a major medical and economic problem in modern society. Nowadays, trauma is the leading cause of mortality in the young aged population [1], [2]. A number of studies report that mechanical trauma can cause direct heart damage, such as coronary artery dissection and cardiac contusion [3], [4]. As a result of advanced prehospital care and regional trauma systems development, fewer critically injured patients are dying at the scene of the accident. However, recently published clinical reports have indicated that mechanical trauma may cause cardiac death even in the absence of direct cardiomyocyte injury during the first 24 h [5], [6], [7], [8]. These results suggest that nonlethal mechanical trauma can induce delayed cardiac injury. However, the mechanisms responsible for nonlethal mechanical trauma-induced delayed cardiac injury have not yet been identified.

There are two main reasons for delayed cardiac injury, including myocardial cell apoptosis and homeostasis [9]. Studies have shown that apoptosis may contribute to cardiac dysfunction, and the inhibition of apoptosis by a variety of pharmacological inhibitors or genetic strategies results in smaller infarction, improved cardiac function, and attenuated cardiac remodeling [10], [11], [12], [13]. Our previous results revealed that the significant cardiomyocyte apoptosis caused by nonlethal mechanical trauma underlies posttraumatic delayed cardiac dysfunction [14]. However, anti-apoptotic therapy alone cannot completely alleviate the delayed cardiac injury, which indicates that there are possibly other mechanisms of delayed cardiac injury involved by nonlethal mechanical trauma.

Homeostasis maintenance is crucial to ensure the function of body organs, and homeostatic dysregulation may cause multiple organ dysfunctions. There is compelling evidence that autophagy is important for the maintenance of homeostasis under basal conditions [15]. Autophagy is an important cellular function that enables the recycling of long-lived proteins or damaged organelles [16]. Autolysosomal degradation of membrane lipids and proteins generates free fatty acids and amino acids, which can be reused to maintain mitochondrial ATP production and protein synthesis and promote cell survival. Disruption of this pathway prevents cell survival in diverse organisms. Studies have shown that autophagy is involved in various physiological processes, such as neurodegenerative diseases, cancer, heart disease, aging, and infections [17], [18], [19], [20]. Although substantial evidence exists that autophagy plays a critical role in homeostasis maintenance and organ function, whether or not autophagy is changed and contributes to delayed cardiac injury after mechanical trauma remains largely unknown.

Therefore, the aims of the present study were 1) to investigate whether nonlethal mechanical trauma may result in the change of cardiomyocyte autophagy; and, if so, 2) to determine whether myocardial autophagy may contribute to delayed cardiac dysfunction.

## Results

### Traumatic Injury caused Significantly Decreased Myocardial Autophagy

**Figure 1:**
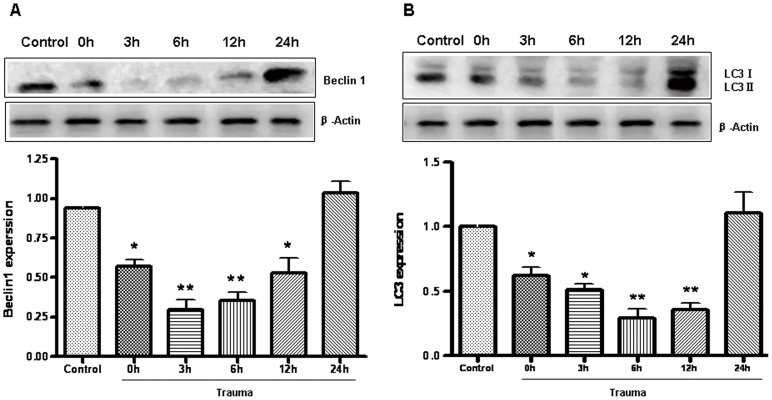
Myocardial autophagy was decreased after nonlethal mechanism trauma. Animals were sacrificed at various times after trauma as indicated. Extracts from injured cardiac were separated on sodium dodecyl sulfate polyacrylamide gel electrophoresis (SDS-PAGE), and protein levels of Beclin 1 (A) and LC3 (B) were detected with immunoblotting. Optical densities of respective protein bands were analyzed with Sigma Scan Pro 5. β-actin was served as the standard. Data are expressed as Mean±SD (n = 6). * p<0.05; ** p<0.01.

**Figure 2:**
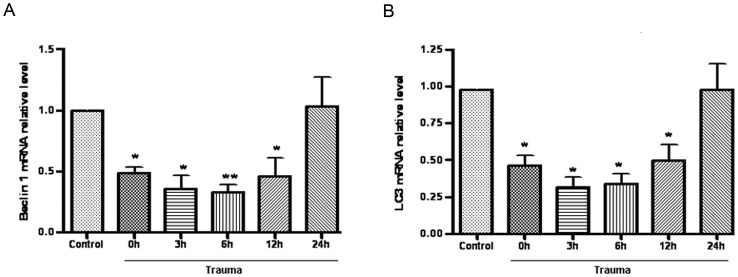
Myocardial autophagy was decreased after nonlethal mechanism trauma. Animals were sacrificed at various times after trauma as indicated. Extracts from injured cardiac were lysised, and mRNA levels of Beclin 1 (A) and LC3 (B) were detected with Real-time PCR. Data are expressed as Mean±SD (n = 6). * p<0.05; ** p<0.01.

To determine how autophagic activity is altered after nonlethal mechanical trauma, the heart was removed at different time points after trauma and the protein levels of Beclin 1 and LC3 were first determined. Beclin 1 (Atg6) is a key protein shown to be involved in the regulation of autophagy [21]. Compared to the sham group rats, Beclin 1 levels were significantly decreased in rats which were killed immediately after nonlethal trauma (time 0), then reached a minimal level at 6 h after trauma, and nearly recovered at 24 h after trauma (Fig. 1A). During the formation of autophagosomal membranes, LC3-I is recruited to the autophagosome where LC3-II is generated by proteolysis and lipidation. Thus, the levels of LC3-II serve as a good indicator of autophagy [22], [23], [24], and the amount of LC3 II is correlated with the extent of autophagosome formation [25]. Similar trends were revealed in the expression of the LC3 II protein, which was decreased shortly after trauma, then reached a minimal level at 6 h after trauma, and nearly recovered at 24 h after trauma (Fig. 1B) compared to sham group rats. To determine whether the mRNA level changed, the mRNA expression of Beclin-1 and LC3 II were evaluated at different time points after trauma. Beclin 1 and LC3 II were decreased at the beginning of the completion of trauma (time 0) and reached a minimal level at 6 h after trauma (Fig. 2A, B) versus sham group rats.

**Figure 3:**
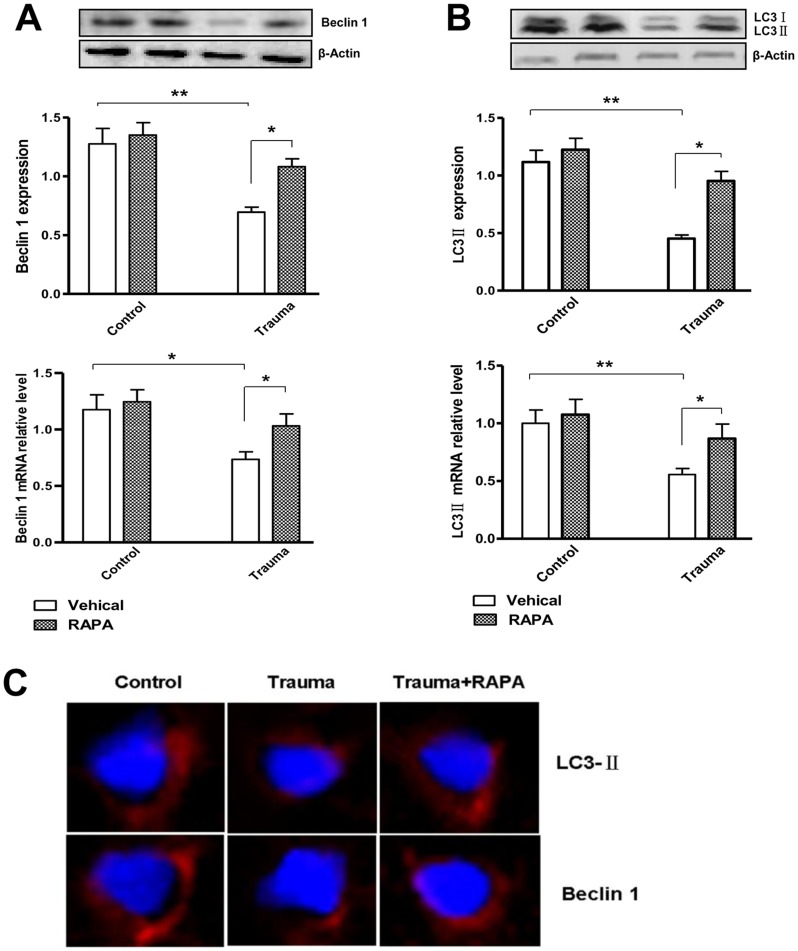
Rapamycin (RAPA) can induce autophagy. After the injection of rapamycin and vecicle 30 min before trauma, the rats were sacrificed at 6 h after trauma. (A and B) The protein and mRNA levels of Beclin 1 and LC3 were detected. Data are expressed as Mean±SD (n = 6). * p<0.05; ** p<0.01. (C) Confocal images of Beclin 1 and LC3. Beclin 1 and LC3 immunoreactivity were present as punctate pot (red) and the nucleus were stained with DAPI (blue). Compared with control-operated animals, trauma group showed decreased staining of Beclin 1 and LC3 6 h after injury. Treatment with rapamycin (RAPA) partially reversed nonlethal trauma-induced down-regulation of staining.

Rapamycin, a macrolide antibiotic, is a widely used inducer of autophagy. To confirm the ability of rapamycin to induce autophagy, rapamycin was intraperitoneally injected 30 min before trauma, and the expression of Beclin 1 and LC3 II were detected 6 h after trauma. Pretreatment with rapamycin significantly induced the mRNA expression of Beclin 1 and LC3 II in the injured myocardium, although administration of this compound had no homodynamic effect in sham animals (Fig. 3A, B). By immunofluorescence staining technology, the Beclin 1 and LC3 II punctate (red) dots were significantly reduced 6 h after trauma, and treatment with rapamycin 30 min before trauma partially prevented trauma-induced alterations in Beclin 1 and LC3 immunoreactivity at 6 h after nonlethal mechanical trauma (Fig. 3C).

### Induction of Autophagy Attenuates Nonlethal Mechanical Trauma-Induced Myocardial Mitochondria Dysfunction

**Figure 4:**
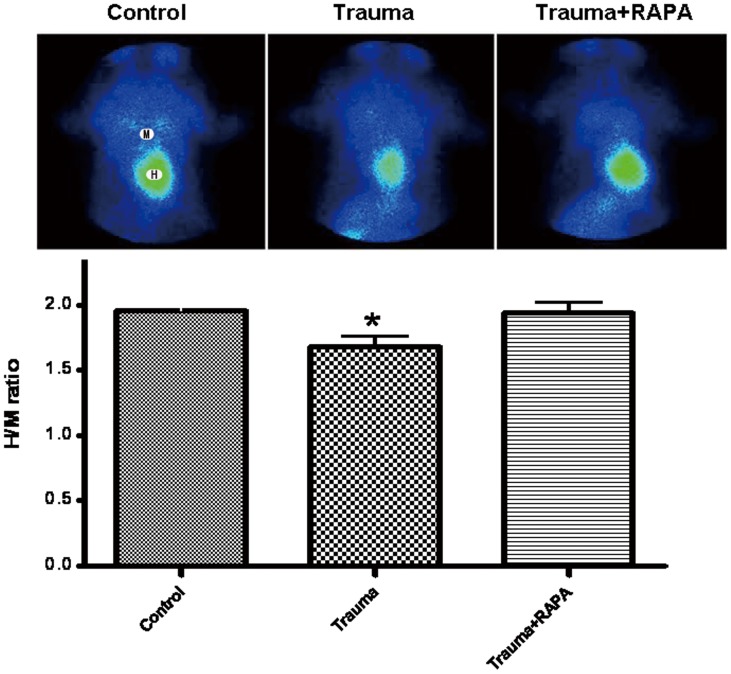
Cardiac radionuclide imaging gained from rats which reflected mitochondrial function. Anterior planar images were obtained 30 min after Technetium 99 m sestamibi (^99 m^Tc-MIBI) intravenous injection. Cardiac ^99 m^Tc-MIBI uptake was expressed as H/M ratio which was obtained using regions of interest positioned over the heart (H) and upper mediastinum (M). Nonlethal mechanical trauma resulted in significant decrease in uptake of ^99 m^Tc-MIBI, which was prevented by administration of rapamycin (RAPA) 30 min before trauma. Data are expressed as Mean ± SD (n = 6). * p<0.05.

It is well known that mitochondria play an especially important role in cardiomyocytes. To evaluate whether nonlethal trauma may cause damage to cardiomyocytes, mitochondria function was evaluated by cardiac radionuclide imaging technology. Cardiac radionuclide imaging is the use of radiopharmaceutical (radioactive forms of certain chemical elements and compounds) together with special cameras known as gamma cameras to obtain information on the function of the heart. It can be used clinically to diagnose and assess various medical conditions. The Technetium 99 m sestamibi (^99 m^Tc-MIBI) is a lipophilic cationic myocardial perfusion imaging agent. Myocardium uptake of ^99 m^Tc-MIBI is dependent on mitochondrial membrane potentials. The distribution of ^99 m^Tc-MIBI in myocardium can be used to reflect cardiac mitochondrial function. Our results showed that cardiac ^99 m^Tc-MIBI distribution was significantly decreased 6 h after nonlethal trauma compared with the sham group. Interestingly, pretreatment with rapamycin 30 min before trauma could increase the distribution of cardiac ^99 m^Tc-MIBI compared with sham group (Fig. 4).

### Induction of Autophagy Attenuates Nonlethal Mechanical Trauma-Induced Cardiac Dysfunction in Isolated Perfused Hearts

**Figure 5:**
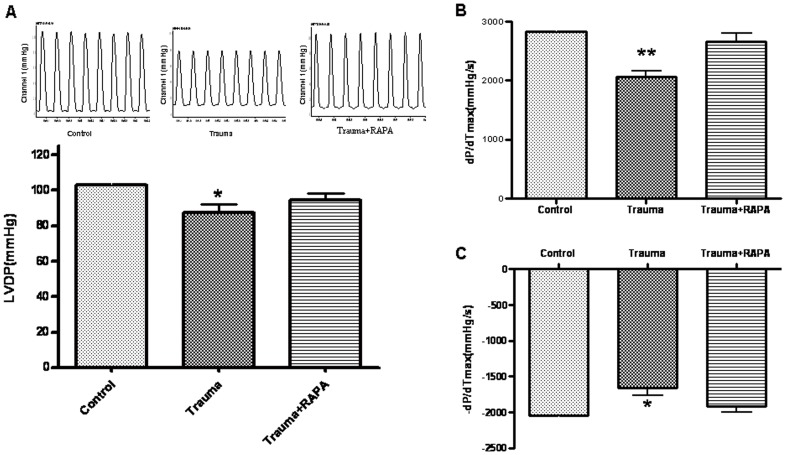
Cardiac function in isolated perfused hearts 24 h after trauma. (A) Nonlethal mechanical trauma resulted in significant decrease in left ventricular developed pressure(LVDP), which was prevented by administration of rapamycin (RAPA) 30 min before trauma. (B and C) Mechanical trauma resulted in significant decrease in the maximal positive and negative values of the instantaneous first derivative of left ventricular pressure (LVP, +dP/dtmax and -dP/dtmax) in isolated perfused hearts 24 h after trauma, which was prevented by administration of RAPA 30 min before trauma. Data are expressed as Mean±SD (n = 6). * p<0.05; ** p<0.01.

Evaluation of myocardial function is the basis for management of heart disease. Our previous studies have shown cardiac function in vivo was normal within 24 h posttrauma [14]. Because the neurohumoral regulation of the cardiac function is important in vivo, a normal cardiac function in vivo does not necessarily indicate that cardiac contractile function is normal. To further determine whether nonlethal trauma may result in cardiac dysfunction when other compensatory factors are eliminated, hearts were isolated 24 h after traumatic injury and perfused in vitro using a Langendorff apparatus. We found that hearts isolated from traumatic rats exhibited a lower LVDP (Fig. 5A), reduced +dP/dtmax, and decreased -dP/dtmax compared with hearts from sham group rats (Fig. 5B, C). Most interestingly, pretreatment with rapamycin 30 min before trauma reversed cardiac dysfunction.

## Discussion

We have made several novel observations in our present study. First, we have demonstrated that nonlethal mechanical trauma resulted in significantly decreased cardiomyocyte autophagy 0 h, 3 h, 6 h, and 12 h after trauma. Second, we have provided direct evidence that the decreased cardiomyocyte autophagy contributes to posttraumatic cardiac dysfunction, and that rapamycin therapy might be an effective strategy to prevent or attenuate postinjury delayed organ dysfunction.

During the formation of autophagosomal membranes, a cytosolic form of LC3 (LC3-I) is conjugated to phosphatidylethanolamine to form LC3-phosphatidylethanolamine conjugate (LC3-II), which is recruited to autophagosomal membranes. LC3-II has been shown to be an autophagosomal marker in mammals. The amount of LC3-II is correlated with the extent of autophagosome formation [26]. Beclin 1, a mammalian homolog of the yeast autophagy-related protein Atg6, was one of the first identified mammalian autophagy proteins, and it plays an important role in bridging autophagy. Therefore, it is commonly used during autophagy detection [27]. In the heart, autophagy occurs constitutively in the normal myocardium but is substantially increased in cases of heart failure, cardiac hypertrophy, and ischemic cardiomyopathy [28], [29], [30]. Inefficient autophagy or its absence causes the myocardium to perform poorly and results in cardiac dysfunction. The functional role of autophagy in heart disease (i.e. whether it mediates cell survival or cell death and whether it up- or down-regulates cellular function) is still poorly understood [24], [31]. We have demonstrated that nonlethal mechanical trauma results in significantly decreased cardiomyocyte autophagy 0 h, 3 h, 6 h, and 12 h after trauma at the mRNA and protein level, and our study focused on whether and how autophagy contributes to posttraumatic cardiac dysfunction.

Autophagy is the primary mechanism for removing damaged organelles, such as mitochondria [32]. Previous studies in mammalian cells have concluded that damaged mitochondria are degraded by autophagy [33]. Insufficient autophagy leads to defective removal of mitochondria [34]. Disruption of the mitochondrial transmembrane potential induces the release of apoptosis-inducing factors, leading to cell dysfunction and death followed by cardiac dysfunction. Intriguingly, myocardial mitochondria function can be detected by cardiac radionuclide imaging, which is a kind of noninvasive detection technology and is widely used clinically to diagnose and assess various medical conditions. Myocardium uptake of ^99 m^Tc-MIBI, a lipophilic cationic myocardial perfusion imaging agent, is dependent on mitochondrial membrane potentials. It is reported that approximately 90% of ^99 m^Tc-MIBI activity in vivo is associated with mitochondria in an energy-dependent manner as a free cationic complex. More than 90% of myocardial ^99 m^Tc-MIBI is localized within the mitochondrial fraction, and retention of ^99 m^Tc-MIBI in the mitochondria relates to mitochondrial function. The lack of redistribution of ^99 m^Tc-MIBI has been shown in experimental and clinical studies. Reverse redistribution of ^99 m^Tc-MIBI has been investigated in patients with acute myocardial infarction and dilated cardiomyopathy [35], [36]. Rapamycin is an FDA-approved antibiotic and immunosuppressant. It inhibits the activity of mTOR (mammalian target of rapamycin) which normally serves as a major negative regulator of autophagy, so it is widely used as an autophagy inducer [37], [38], [39]. Our studies found that myocardial mitochondria^ 99 m^Tc-MIBI distribution was decreased after trauma. Interestingly, pretreatment with rapamycin significantly increased the myocardial uptake of ^99 m^Tc-MIBI, which indicated the improvement of mitochondrial function. These results demonstrated that decreased cardiomyocyte autophagy after trauma is a critical contributor to posttrauma cardiac mitochondrial damage.

Our previous results demonstrated that the nonlethal mechanical trauma failed to induce a typical traumatic shock and the cardiac function in vivo measured 0 to 24 h posttrauma was normal [14]. Because the cardiac function in vivo is regulated by multiple neuronal and humoral factors in vivo, a normal cardiac function in vivo does not necessarily mean that cardiac contractile function is normal. To eliminate other compensatory factors, cardiac function in isolated perfused hearts was detected. The results showed that hearts isolated from traumatic rats exhibited a lower LVDP, reduced +dP/dtmax and decreased -dP/dtmax compared with hearts from sham trauma rats. Pretreatment with rapamycin blocked trauma-induced cardiac dysfunction. These results demonstrated that the decrease of traumatic-cardiomyocyte autophagy may be a critical contributor to cardiac dysfunction in vitro.

Studies have shown that mitochondrial dysfunction may be a major contributor to multiorgan failure [40], and in our study, we were interested in understanding whether there is a link between decreased mitochondrial function and cardiac dysfunction in isolated perfused hearts induced by nonlethal trauma.

The heart needs a steady supply of energy to function properly. Maximal cardiac work, which requires higher rates of energy supply to myofibrils and oxygen consumption, is the most affected parameter. Peak dP/dtmax has historically been used as an index of maximal cardiac work that is not influenced by afterload. Mitochondria are the major source of cellular energy, providing more than 90% of the ATP required for cell work. It is generally considered that mitochondria regulate cardiac cell contractility by providing ATP for cellular ATPases. When mitochondria are rendered dysfunctional, a bioenergetic limitation of cell and cardiac function is expected. Maintenance of energy control depends on the structural integrity of the mitochondrion. Disruption of the mitochondrial transmembrane potential causes electron transport to become uncoupled from ATP synthesis. Under these conditions, ATP synthesis is inhibited. In our present study, we found that mitochondrial transmembrane potential was damaged and +dP/dtmax of the isolated heart after trauma decreased dramatically, whereas pretreatment with rapamycin could partly reverse this process, which indicates that the declined mitochondrial function may be contributed to the cardiac dysfunction of the isolated heart after nonlethal trauma.

In summary, in the present study we demonstrated for the first time that myocardial autophagy expression decreased in nonlethal mechanical trauma, and decreased autophagy contributed to mitochondrial dysfunction and cardiac injury. Moreover, mitochondrial dysfunction may be relevant to cardiac dysfunction in nonlethal trauma, but this requires further study to provide a more definitive answer. Though our study has some limitations, it strongly suggests that cardiac damage induced by nonlethal mechanical trauma can be detected by noninvasive radionuclide imaging, though it was detected normally by common clinical examination, such as ECG, myocardial enzymes, echocardiography and X-ray. Furthermore, induction of autophagy may be a novel strategy for reducing posttrauma multiple organ failure. In addition, whether there is a more sensitive target protein to detect heart damage after nonlethal mechanical trauma is a key concern.

## Materials and Methods

### Ethics Statement

This study conforms to the Guide for the Care and Use of Laboratory Animals published by the US National Institutes of Health, the Guide for the Care and Use of Laboratory Animals’ protocol, published by the Ministry of the People’s Republic of China (issued 3 June, 2004), and approved by the Institutional Animal Care and Use Committee of Shanxi Medical University. All Wistar rats were performed under 10% chloral hydrate anesthesia, and all efforts were made to minimize suffering.

### Nonlethal Traumatic Rat Model

Noble-Collip drum exposure is a well-accepted traumatic model that results in a whole body nonpenetrative mechanical trauma. In brief, male adult C57B16/J rats were anesthetized with 10% chloral hydrate (300 mg/kg ip). Then the rats were placed in a Noble-Collip drum and subjected to a total of 5 min rotations (200 rotations at a rate of 40 r/min). Traumatic rats were injured when the wheel was rotated, while sham trauma rats were taped on the inner wall of the drum to avoid traumatic injury. Nonlethal mechanical trauma rat models used in this experiment by the Noble-Collip drum is characterized by the lack of circulatory shock (mean arterial blood pressure >75 mmHg within 24 h after trauma), no direct cardiac injury (i.e., cardiac contusion and pericardial bleeding), and a 100% 24 h survival rate [14].

### Western Blot

In brief, cardiac tissue (100 mg) was lysed in RIPA buffer (Thermo scientific, 89900) and sonicated. Protein concentrations were measured by BCA (Thermo scientific, 23228). Proteins were resolved by standard SDS-PAGE and transferred to polyvinylidene difluoride (PVDF) membranes (Whatman, 10485289), followed by detection with the primary antibodies–anti-Beclin 1 monoclonal (1∶1,000; Santa Cruz, sc-48381), anti LC3 II monoclonal (1∶1000; Cell Signaling Tech, 2775) or anti-β-actin monoclonal (1∶1,000; Cell Signaling, 2118)–followed by a secondary antibody (IRDye800CW, 926-32210). The fluorescence density on PVDF membranes were detected and analyzed with a LI-COR imaging system (LI-COR Biosciences, 9201-01).

### Real-time PCR

Analysis of gene expression was studied using Real-time quantitative PCR with SYBR Green (Sigma, S9430) detection in the Real-time PCR System (Stratagene, Mx3005). Total RNA was extracted from the cardiac tissue with Trizol reagent (Invitrogen, 15596-026). 3 µg of total RNA was reversely transcribed into cDNA. The thermal profile for SYBR Green PCR was according to standard protocol as follows: 1 cycle of 95°C for 5 sec; 40 cycles of 95°C for 5 sec, 60°C for 20 sec; and a final extension at 95°C for 1 min, 55°C for 30 min, and 95°C for 30 s. The primer sequences were as follows: LC3 II, sense: 5′-CAT GCC GTC CGA GAA GAC CT-3′ and antisense: 5′-GAT GAG CCG GAC ATC TTC CAC T-3′ (GenBank TMaccession number, NM022867.2); Beclin 1, sense: 5′-TTG GCC AAT AAG ATG GGT CTG AA-3′ and antisense: 5′-TGT CAG GGA CTC CAG ATA CGA GTG-3′ (GenBankTM accession number, NM001034117.1). Samples were normalized against β-actin expression (sense: 5′-GGC TAC AGC TTC ACC ACC AC-3′ and antisense: 5′-TCA GGA GGA GCA ATG ATC TTG-3′, GenBankTM accession number, NM031144) to ensure equal loading. The specificity of the amplified product was monitored by its dissociation curve. The results, expressed as the fold difference in the number of LC3 or Beclin 1 copies relative to the number of β-actin gene copies, were determined by the relative quantitative 2-ΔΔCt method. ΔΔCt = ΔCt (target gene)−ΔCt (β-actin) and ΔCt (target gene) = Ct (experimental-target)−Ct (control-target) and ΔCt (β-actin) = Ct (experimental-β-actin)−Ct (control-β-actin).

### Immunofluorescence

Myocardial tissue samples were embedded in JUNG Tissue Freezing Medium (Leica Mycrosystems Nussloch GmbH, 020108926) and were sectioned at 10 µm thickness with cryostat (Leica Microsystems Nussloch GmbH, CM1850), air-dried for 60 min, fixed with acetone for 15 min at 4°C and stored at −20°C until use. After blocking with Immunol Staining Blocking Buffer (Beyotime Biotech, P0102) for 1 h, sections were incubated with antibodies against LC3II (1∶400; Cell Signaling Tech, 2775) and Beclin 1 (1∶50, Santa Cruz Biotech, sc-48341) in a humidified container at 4°C overnight. The sections were rinsed three times with PBS and sequentially incubated with tetramethylrhodaminyl isothiocyanate (TRITC)-conjugated secondary anti-rabbit IgG (1∶50, Beijing Zhongshan Golden Bridge Biotechnology, ZF-0316) or TRITC-conjugated secondary anti-mouse IgG (1∶50, Beijing Zhongshan Golden Bridge Biotechnology, ZF-0313). After washing three times with PBS, 2-(4-Amidinophenyl)-6-indolecarbamidine dihydrochloride (DAPI, Beyotime Biotech, C1005) the solution was added to stain the cell nucleus for 3 min. Sections were then washed in PBS and sealed with a coverslip. The slides were analyzed with a laser confocal microscopy (OLYMPUS, FV1000).

### Preparation of ^99 m^Tc-MIBI and Myocardial Imaging

1110 MBq of ^99 m^Tc0_4_^−^ was added to the Cu(MIBI)_4_BF_4_ (E.I. Shihong, Products Division, X-06-2) and it was then heated in a water bath at 100°C for 10 min. After cooling, the appropriate amount of solution for 185 MBq of activity was withdrawn into a lead-shielded syringe. Thin-layer chromatography and Sep-pak analyses were performed on the Technetium 99 m sestamibi (^99 m^Tc-MIBI) compound to evaluate the purity of the radiopharmaceutical. The ^99 m^Tc-MIBI compound chromatographed with an Rf of 0.54. In the Sep-pak analysis, over 99% of the ^99 m^Tc-MIBI compound remained in the cartridge. An 18.5-MBq dose of ^99 m^Tc-MIBI was injected slowly through the rat tail vein. The planar imaging was obtained approximately 30 minutes after injection. Images of the heart were obtained with a conventional gamma scintillation camera (Mobile Radioisotope Camera, Model BHP6602) interfaced with a mobile nuclear imaging computer system. A low-energy high-resolution pinhole collimator was used, and a 20% energy window encompassing the 140 keV photopeak was selected. Imaging was performed projection with the chest open. Images were collected in a 256×256×16 matrix format.

### Cardiac Function in the Isolated Perfused Heart

At 24 h after trauma, animals were re-anesthetized with 10% chloral hydrate (300 mg/kg ip) and heparinized with sodium heparin (1,000 U/kg ip). Ten minutes after heparin injection, hearts were rapidly excised from deeply anesthetized rats and placed immediately into an ice-cold Krebs-Henseleit bicarbonate solution containing (in mM) 118 NaCl, 25 NaHCO3, 4.7 KCl, 1.2 MgSO4, 1.2 KH2PO4, 2.25 CaCl2, 0.5 EDTA, and 11.1 glucose. Within 30 s, the heart was mounted onto a Langendorff heart perfusion apparatus (Radnoti Glass Technology, 800-428-1416). The heart was perfused in a retrograde fashion via the aorta at a constant pressure of 70 mmHg with Krebs-Henseleit solution oxygenated with 95% O2-5% CO2 to maintain pH 7.4 at 37°C. Hearts were paced at 260 beats/min with a Grass Stimulator and coronary flow (CF) was measured via an in-line flow probe connected to an ultrasonic flow meter (Transonic Systems, T206). All hearts were equilibrated for 30 min.

To assess contractile function, a latex balloon was inserted into the left ventricular cavity through the mitral orifice and connected to a pressure transducer (Lakewood, CO 80215). The balloon was initially inflated with saline to produce an end-diastolic pressure of 8 to 10 mmHg, which is on the plateau of the Starling curve for this preparation. Left ventricular pressure (LVP) and CF were continually recorded via a data acquisition system (PowerLab; ADInstruments, 4/30). The left ventricular (LV) systolic pressure (LVSP), LV end-diastolic pressure (LVEDP), LV developed pressure (LVDP = LVSP−LVEDP), the maximal positive and negative values of the instantaneous first derivative of LVP (+dP/dtmax and -dP/dtmax) were obtained by using computer algorithms and an interactive videographics program (PowerLab, Chart V5.0 for Windows).

### Statistical Analysis

Data were presented as mean ± standard deviation (Mean±SD). Statistical comparisons between groups were performed by the use of one-way analysis of variance (ANOVA). Statistical significance was set at *P*<0.05.
